# Implementation of an HIV Prevention Intervention at Historically Black Colleges and Universities and Predominantly Black Institutions

**DOI:** 10.3390/ijerph21111395

**Published:** 2024-10-23

**Authors:** Rhonda C. Holliday, Samantha D. Martin, Romell Phillips, Zahra Shahin, Kourtnii Farley, Alyssa B. Cahoy, Terry Ross

**Affiliations:** 1 Department of Community Health and Preventive Medicine, Prevention Research Center, Morehouse School of Medicine, Atlanta, GA 30310, USA; sammartin@msm.edu (S.D.M.); rphillips@msm.edu (R.P.); zshahin@msm.edu (Z.S.); kfarley@msm.edu (K.F.); 2 Office of Strategic Research Communications, University of Houston Division of Research, Houston, TX 77004, USA; acahoy@uh.edu; 3 Prevention Research Center, Community Coalition Board Member, Morehouse School of Medicine, East Point, GA 30344, USA; t-ross01@hotmail.com

**Keywords:** HIV prevention, Black Americans, young adults, college campuses, HBCUs, qualitative, implementation

## Abstract

Black Americans and the Southern United States are disproportionately represented in the HIV epidemic. Historically Black Colleges and Universities (HBCUs) and Predominantly Black Institutions (PBIs), often located in communities that have been historically underserved, are uniquely positioned to implement HIV prevention interventions focused on Black young adults. The purpose of the current study was to conduct a qualitative study, using the Consolidated Framework for Implementation Research (CFIR) model as a guide, to identify the barriers and facilitators to implementing an HIV intervention pre- and post-implementation. Pre-implementation key informant interviews with administrators, faculty, and staff, alongside focus groups with students, highlighted several themes as potential influencers on intervention rollout. These included perceived need, campus health resources, cost, personnel availability, student priorities, HIV-related stigma, and institutional culture. Post-implementation interviews with campus liaisons further revealed themes including institutional culture, external partnerships, internal communication, student health resource accessibility, and peer educator recruitment and retention. These findings underscore the complexities of implementing public health interventions in academic settings and may guide future efforts at HBCUs and PBIs to effectively address HIV prevention.

## 1. Introduction

The HIV epidemic disproportionately impacts Black Americans and those living in the South. According to the Centers for Disease Control and Prevention (CDC), Black Americans accounted for 40% of the 32,100 new HIV cases in 2021, despite only representing 12% of the general population [1]. Adolescents and young adults aged 13 to 24 represented 19% of new HIV diagnoses, with Black young adults comprising 53% of those diagnoses [2]. Despite declining HIV infection rates in the U.S., college-aged Black Americans continue to be at a higher risk of acquiring HIV compared to the general population. When examining the impact of the HIV epidemic geographically, 52% of new HIV infections in 2021 were found in the Southern United States, and 41% of the approximately 1.2 million people living with HIV in the U.S. live in the South, despite the South only accounting for 38% of the U.S. population [1,3,4]. Furthermore, as of 2022, 56% of Black Americans live in the South [5]; thus, the disproportionate rates of HIV among Black Americans should not be unexpected in the South. 

There are a multitude of systemic factors that contribute to the prevalence of HIV in the Black community in the Southern U.S., including racial inequality and bias, HIV-related stigma, poverty, healthcare access, and a larger occurrence of sexually transmitted infections (STIs) [6]. Additionally, general mistrust of healthcare institutions due to medical discrimination may deter Black students from engaging in HIV preventive services [7]. Given this context, it is important to identify and implement structural interventions to address HIV prevention that are specifically designed to reduce the burden of HIV among Black Americans living in the South. Structural interventions on college campuses that prioritize Black students may offer an opportunity to impact the trajectory of new HIV cases among Black Americans in the Southern United States. Historically Black Colleges and Universities (HBCUs) are an often-overlooked arena to address the HIV epidemic [8]. Despite their historical underfunding and location in healthcare deserts [9], implementing interventions in these settings may prove particularly useful for ending the HIV epidemic.

### 1.1. Historically Black Colleges and Universities Setting

HBCUs and Predominantly Black Institutions (PBIs) provide a unique opportunity to address HIV prevention among Black young adults. HBCUs were born out of the need to provide access to higher education to Black citizens during a time when they were not permitted to attend established educational institutions due to segregation [10]. These institutions are typically viewed as paternal, while also being viewed as supportive protective environments for Black students [11,12,13,14]. In 2022, there were roughly 343,700 students enrolled at 101 HBCUs across the United States [15]. There are HBCUs in 21 states, along with the District of Columbia and the Virgin Islands, and they are mostly clustered in the South [16]. HBCUs in the South are often located in some of the most underserved areas (both urban and rural), which also experience high rates of poverty, income inequality, and HIV [17,18]. According to LeBlanc et al., [17] 10 of the 14 states with the highest burden of HIV were situated in the South and had four or more HBCUs. Sutton et al. [18] found that 96% of HBCUs were in 69 southern counties, and those counties had higher rates of HIV among Black people, with 80% of those counties having rates that were disproportionately higher than those of whites. Sutton et al. [18] advocates for innovative strategies to address HIV/AIDs in the South by developing and strengthening relationships between HBCUs and public health institutions to reach communities that are disproportionately affected by HIV/AIDS.

### 1.2. HIV Risk Behaviors Among Black Students at HBCUs 

Studies have found that inconsistent condom use, substance use during sex, and multiple sexual partners may increase the risk of HIV transmission among Black students attending HBCUs [6,19,20,21,22]. HIV testing remains an important HIV prevention strategy. There have been several studies that have examined HIV testing rates, perceptions, and barriers to HIV testing on HBCU campuses. In one such study, Payne et al., [23] found that among students who were approached about rapid HIV testing, 50% agreed to be tested and 48% of the participants reported being previously tested for HIV. Barriers to HIV testing included the perception that receiving an HIV test could affect the relationship with one’s partner, lack of awareness of HIV testing sites, and fear that there would be no confidentiality regarding the results of the HIV test. In another survey of HBCU students, 77% of participants declined HIV testing when offered, and 30% were unaware of their HIV status [24]. Although both men and women appeared to be equally knowledgeable about HIV, men were more likely to be tested when testing was made available [24]. Holliday et al. [25] reported that in a study of nearly 2400 people, nearly one-third of HBCU students and approximately 18% of community members in the study had not been previously tested for HIV. Thomas et al. [26], in a study of HIV testing on seven HBCU campuses, found that 42% of students had never been tested for HIV. Finally, Marshall [6] found that of 615 HBCU students surveyed, 27.2% reported having been tested at least once, while 42% had never been tested. Results also indicated that 48% of men reported having never been tested, in contrast to 39% of women. Of those who had been tested for HIV, slightly more than half (51%) indicated they had discussed their status with their partners.

### 1.3. HIV Structural Interventions at HBCUs

Structural interventions that prioritize HBCUs are essential to addressing health issues of young Black people, although the implementation of those interventions may face unique challenges as well as facilitators. In a survey of 105 HBCU campuses, Braithwaite and our team [27] sought to determine the availability of condoms and the presence of condom-dispensing machines. Of the respondents for this study, 85.7% provided condoms at their institutions, and only 11.2% used condom dispensing machines. The most common place for condoms at HBCUs was in the student health centers, similar to results from Butler et al. [28]. Condom dispensing machines, while potentially providing more anonymity when acquiring condoms, were also subject to vandalism of the machines and the condoms themselves, as well as theft of condoms.

In 2024, Downer [29] sought to effect change regarding HIV prevention on HBCU campuses through health centers. The HBCU-HIV Prevention (H2P) Project was designed to address provider discomfort with prescribing and advising young adults about PrEP through the implementation of an educational intervention to increase provider knowledge and awareness of PrEP. The study recruited 237 providers working in health centers at HBCUs across the country. The H2P intervention improved provider knowledge between 17 and 24%, and three months post-intervention, the providers were using the knowledge gained in their communication about PrEP [29]. Other interventions on HBCUs have included HIV testing and linkage to care [6,30], HIV education [25,31,32], HIV awareness media campaigns [33], and technology and social media campaigns [34]. 

### 1.4. Take CHARGE

Take CHARGE was a structural intervention designed to implement three evidence-based strategies: HIV testing, HIV education, and condom distribution to address HIV among emerging Black adults. The main goal of the project was to create and implement tailored interventions that considered the cultural norms of emerging Black adults on college campuses using community-engagement strategies. The study was conducted on the campuses of three HBCUs and one PBI located in urban and rural areas of a southeastern state heavily impacted by the HIV epidemic. The current study sought to identify the pre- and post-implementation barriers and facilitators to implementing an HIV prevention intervention on these campuses using qualitative methods. The study received Institutional Review Board approval from the lead institution where the study team was housed, and from two participating institutions. The remaining institutions deferred IRB review to the lead institution. 

## 2. Materials and Methods

### 2.1. Qualitative Data Collection

Qualitative data were collected before the implementation of the intervention activities (2020) and at the conclusion of the intervention activities (2024). Pre-implementation data collection included key informant interviews and focus groups, while post-implementation data collection included only key informant interviews. The key informant interview and focus group guides were developed using the Consolidated Framework for Implementation Research (CFIR) [35]. All data collection was conducted virtually using the Zoom platform, allowing for video and audio recording of all qualitative interviews. Participants were provided with the option of having their cameras on or off during data collection. The interviews and focus groups lasted 60 to 90 min and included a moderator and at least one notetaker. Recordings were saved to the hard drive of a password-protected computer and were later transcribed. 

### 2.2. Participants

Eligible participants for the key informant interviews included faculty, staff, and administrators at participating institutions (see Table 1). Discussions centered around the structural characteristics of the institutions, external partnerships, student needs and resources, and the costs associated with implementing the intervention.

For three of the four institutions, the initial interviews were conducted with staff from the student health clinics. Those staff then identified other staff, faculty, and administrators to participate. The fourth institution did not have a student health clinic; therefore, we identified an administrator for the initial interview who subsequently identified faculty and counseling center staff to participate. The selection of key informants (n =17) was based on their roles within the institution and their potential influence on HIV prevention initiatives. After implementation (n = 6), campus liaisons who managed daily activities on each site and representatives from community partners who assisted with implementation activities were recruited to participate in additional key informant interviews. A total of 23 key informant interviews were conducted (see Table 1). Each key informant received an electronic consent form and consent was obtained before the interview. A USD 100 gift card was offered as an incentive for participating in the interview.

Focus group participants were Black students aged 18 to 24 attending the participating institutions (see Table 2). Separate focus groups were held for each campus. Key informants identified student leaders to help disseminate information about the focus groups. Student leaders were provided flyers that were distributed via social media and posted in dorms. Due to COVID-19 regulations that restricted campus movement and access, social media and posting in dorms were deemed the best ways to recruit students. 

There were seven focus groups comprised of four to eight students at participating institutions. Forty students participated in the seven focus groups (see Table 2). The focus groups were structured to encourage open dialogue, allowing participants to share their experiences and viewpoints in a relaxed setting. The research team utilized a semi-structured guide with open-ended questions to ensure that key topics were addressed while leaving room for organic discussion. The focus groups aimed to gather student perspectives on various aspects of sexual health and about implementing Take CHARGE on their campuses. Consent was obtained, via electronic consent, prior to joining the focus group. Additionally, participants were provided a summary of the study, allowed to ask questions, and given the opportunity to leave the focus group prior to the start of the recording. A USD 50 gift card was offered as an incentive for participation. Focus groups were conducted before implementation. Post-implementation focus groups were not conducted due to recruitment issues. 

### 2.3. Qualitative Data Analysis

The recordings were transcribed by members of the study staff and a local transcribing service. Analyses of the transcripts were conducted using Rapid Assessment Response and Evaluation (RARE) methodology. RARE is a systematic rapid approach to analyzing qualitative data that uses data reduction techniques to find the main points of the data. RARE is particularly useful when addressing urgent or rapidly evolving situations, allowing researchers to adapt to changing contexts [36].

A coding template was developed based on the domains outlined in the CFIR model (2009 version), which provided a structured foundation for analyzing the collected data from the key informant interviews and focus groups. Initially, a subset of the data was reviewed to identify key themes and patterns that aligned with the CFIR domains. Using this review as a guide, a coding framework consisting of a set of predefined codes corresponding to these domains was established. This framework allowed for consistent categorization of the data across all transcripts.

Data were managed and coded in Dedoose, ref. [37] a qualitative data analysis software that facilitates the application of codes and the organization of data for analysis. All transcripts were coded according to the coding framework. The coded data were compiled into a matrix, with rows representing individual focus groups or interviews and columns representing the CFIR domains (codes). This matrix structure provided a clear visual representation of the data, allowing for quick identification of patterns, themes, and variations across the different data sources. The matrix was used to guide the analysis, enabling the study team to examine the distribution of codes and identify relationships among them. This approach facilitated the identification of common themes and emerging insights on barriers and facilitators to implementing HIV prevention on college campuses (for a full list of themes and their corresponding CFIR domains, see Table 3). To ensure the reliability of the coding process, multiple members of the study staff participated in the coding and analysis, allowing for cross-checking and consensus-building on the application of codes. Additionally, RARE analysis allowed for iterative adjustments to the coding framework and matrix as new insights emerged during the analysis. Senior research staff and the study project director trained the study staff on RARE and thematic analysis methodology. Coding was supervised by the study project director and senior research staff and was conducted by graduate and undergraduate research assistants. Data were then analyzed by the principal investigator, project director, and graduate research assistants.

The study team for this project consisted of individuals with diverse professional and academic backgrounds, including in public health, sociology, and higher education. Most of the coders identified as Black and have either attended or worked at PBIs and/or HBCUs, which provides us with a deeper contextual understanding of the challenges faced by these institutions. However, we acknowledge that this shared experience may also introduce potential biases, particularly in how we perceive institutional efforts to address HIV prevention and student engagement. To mitigate this, we engaged in regular discussions to ensure that our analysis was grounded in the data by consistently verifying that any conclusions drawn were supported by participant responses, rather than shaped by our personal experiences.

## 3. Results

### 3.1. Pre-Implementation

Pre-implementation data analysis revealed several barriers and facilitators for implementing Take CHARGE. After mapping the data to CFIR domains, several themes emerged. Themes included the perceived need for the intervention, dedicated campus health resources, limited personnel, cost concerns, external partnerships, institutional culture, student access to health resources, student priorities, and navigating stigma (see Table 3).

It is important to note that pre-implementation data collection occurred between 2020 and 2021 as institutions were experiencing unprecedented circumstances due to the COVID-19 pandemic. Consequently, institutional priorities shifted to focus on reducing the spread of COVID-19. This directly impacted the priorities of student health services:

“*So the priority of the institution is to make sure the students are safe. … We are doing new student orientation. we usually do something around HIV/STIs, prevention, condom distribution... I need to talk to [senior administration] about that because [we’re] just concerned with COVID.*”

(Administrator)

#### 3.1.1. Perceived Need for the Intervention 

Consistent across key informant interviews and focus groups was the perceived need for the implementation of Take CHARGE at the participating campuses. All key informants were in support of the intervention and suggested it would not be difficult to implement:

“*The project to me sounds … like it would be a good fit for the institution, and it sounds like our students could really benefit from a project like this on our campus.*”

(Administration)

Students also felt that there was a need to increase HIV prevention activities on campus. While students were aware of some resources for HIV awareness and testing on campus, students expressed a lack of information available and open discussion regarding sexual health and HIV prevention in the campus community:

“*I was particularly interested in this project especially trying to get more information about HIV, STD, health, aid, and all that on campus, since I don’t think it’s been readily available…*”

(Focus group)

#### 3.1.2. Dedicated Campus Health Resources

Participating institutions had varying dedicated resources to implement HIV and substance use prevention services. For example, one school lacked a student health services center, another had a robust student health services center, while the remaining campuses had recently decreased the capacity of their student health services and were transitioning to a shared student health services center with a neighboring HBCU.

This transition to a shared student health services center was in its early stages during the pre-implementation data collection and was largely viewed as a barrier. Additionally, this transition occurred at the beginning of the COVID-19 pandemic, when health services at all institutions were primarily focused on keeping students safe during the pandemic:

“*We’re actually getting ready to change. We’re not going to be a clinic anymore...-[the school] is going to contract where we would--the students got to go up the hill and get medical services.*”

(Student Health Services Staff)

Despite the variation in dedicated campus resources, several key informants suggested that they would still be able to implement the intervention. This determination also highlights how HBCUs are accustomed to operating with limited resources:

“*And so it wouldn’t be difficult to implement this at all. Um, I don’t think that it would be, um… I think just as we implement everything else, we will implement this as well. I don’t think it will be on a scale of, you know, ‘we’ll implement it later’ or ‘We got something more important to do now.’ I think once we get started, we just get started.*”

(Student health services)

#### 3.1.3. Personnel

A primary concern among key informants was the staff and administrative capacity to implement Take CHARGE. Respondents at all institutions expressed concerns about limited or reduced personnel to conduct additional programming. With already limited staff, student health services had fewer resources to dedicate to HIV prevention programming:

“*I’m trying to turn my attention more towards being proactive and trying to utilize more partnerships and off campus resources because after this year, I have one full time therapist and one part time therapist and it’s just not—we’re not enough for the whole campus.*”

(Student Health Services)

“*One of the things that suffered is our is our outreach efforts, because we just were swamped with students who were coming through the door that it’s hard for us to get out onto the campus and do that sort of work.*”

(Student Health Services Staff)

Despite limited personnel, key informants suggested that staff, faculty, and administrators would support the intervention if it could be integrated into their existing work:

“*I’m happy to assist with anything that doesn’t require a whole lot of my time. So if this could somehow be integrated into work that I’m already doing, even better, you know.*”

(Student health services)

#### 3.1.4. Costs 

The cost of implementing HIV prevention activities was a concern for all institutions. Several key informants discussed concerns regarding the ability to pay for increased HIV prevention programming due to limited institutional and/or departmental budgets. For example, one administrator stated that budgetary constraints limited the ability to increase access to HIV testing:

“*I know what I would like to see if I didn’t have budgetary constraints is for students to have a way to get tested when they want to get tested.*”

(Administration)

Some key informants suggested integrating intervention activities with other health initiatives to leverage pre-existing fiscal and human resources:

“*As a part of this mental health initiative that I was sharing with you, we received quite a bit of funding from the university system. Some of that money is allocated for peer training. I think that would be an opportune time for us to start that peer counseling program.*”

(Student Health Services Staff)

Key informants also suggested that some costs for HIV testing and condom distribution were mitigated through donations from external organizations:

“*It costs each school about $100,000 for the tests. But we didn’t pay that. [The external partner] wrote that off and provided it to us at no cost…*”

(Administration)

“*We get condoms donated from so many different sources. So we actually do a campaign around getting condoms from different vendors, as well as making sure the students have whatever condoms they want.*”

(Student Health Services)

#### 3.1.5. Institutional Culture

All institutions were described as religious or conservative. However, respondents did not believe that this would impact the intervention and focused on the supportive environment of the institution:

“*I think at the more macro level, it’s probably a social-conservative leaning institution. but I think at the micro level with the students themselves and the faculty it is becoming more progressive.*”

(Administration)

Conversely, students indicated a lack of open discussion regarding HIV and sexual health that contributed to a lack of preventative sexual health education, with students not receiving information about HIV or STIs until after they or a close loved one tested positive:

“*In all honesty, I don’t think that we get the information until it’s too late or happens to us, or someone close that we know. I know that’s how I found out more about it, if I may be truthful.*”

(Focus group)

While some students felt comfortable getting tested for STIs on campus, others suggested that stigmatizing attitudes toward sexual activity might discourage students from seeking testing. Several students mentioned that statements from faculty, staff, and administration about sexual health often focused more on preventing pregnancy, than on providing comprehensive and supportive discussions about sexual health. One participant recalled another student sharing an experience they had at student health services:

“*I know like if you’re taking a STI test, then it’s recommended that you take a pregnancy test as well, and I feel like some people would go and they’ll feel like the nurse is shaming them. Like they’ll make a comment like oh you better not be pregnant. So, that’s like discouraging some people. Like you wouldn’t—like you shouldn’t experience that when you’re going to like take a test.*”

(Focus group)

Despite this socially conservative-leaning culture, key informants suggested that close, caring relationships existed among students and campus personnel, consisting of informal open-door policies and open communication. Key informants felt school cultures prioritized meeting student needs and providing them with the resources and support they needed:

“*You know students, I would say almost the entire college, has my cell number. So that type of level of accessibility to [senior leadership] is not common at most schools. So--and it’s not just me. It’s also [other senior leadership] as well. Students have access to and it’s everybody down the line. So we don’t look at them as our customers, they actually are our family.... Our very close-knit family as it pertains to student services at [the college].*”

(Student services administration)

While some focus group participants felt that staff members were judgmental regarding sexual health, others felt that student health services staff were supportive and non-judgmental when it came to HIV testing:

“*I think the people that are very welcoming, oh, they don’t really judge you. It’s just like, hey, come get tested. And it’s like, Okay, cool. Yeah, so I think that they’re welcoming, but I think that accessibility is the main thing.*”

(Focus group participant)

#### 3.1.6. Student Access to Health Resources

Students may rely more heavily on health resources provided by their college or university due to a lack of transportation to facilitate access to community resources:

“*Many of our students are first-generation college students and don’t have a lot of access to healthcare and this type of education.*”

(Administration)

“*I wish it was more available on a more regular basis for students, and then it would be more accessible. Because see not a lot of our students have transportation because we live in the South and transportation is a big, huge issue, so they can’t necessarily get to these places, especially if it is after school hours… Most classes end at 5:00, so then your transportation services are closed.*”

(Administration)

While key informants were aware that their schools were positioned to fill the gap in health resources, this was not reflected in student perceptions about HIV services and programming. Students expressed a desire for increased access to HIV prevention programming on campus. Students suggested that existing services were insufficient, and mentioned that they were not always aware of where or when services could be accessed:

“*So, yeah, it really does need to be more accessible. Because two days out of the week is just not you know, it’s not cutting it for a lot of people. And I forgot what the two days even are. But even if they could have like, an event, like a month, that’s it, the it’s hosted somewhere else or something like that…*”

(Focus group participant)

Students were also enthusiastic about receiving information or testing from community-based organizations. Representatives from CBOs were viewed as trustworthy and reliable sources that care about their communities:

“*I know the school has done a couple of partnerships with a couple of other organizations before…. What I like about that is you take the institution of learning, and you partner that with groups who are trying to make more people aware who understand the knowledge that they’re trying to give to people. And I just kind of feel like, okay, this is an organization that’s dedicated to spread positive information awareness about this topic. I trust that they aren’t going to spread mal-information…and I trust the school… So, it’s people in an organization I trust mixed with an environment that I’m familiar with that works*”

(Focus group participant)

#### 3.1.7. Student Priorities

The utilization of health resources was also influenced by student priorities.

While key informants believed that students would generally be receptive to the intervention, respondents also suggested that conflicting student priorities may impact their participation in intervention activities, and therefore implementation design and recruitment should be mindful of student priorities:

“*College life has so much so many offerings and if there’s a frat party going on at this time and or something else that is more fun and then you try to throw in some health promotion stuff is not gonna fly, they will— that would definitely be a barrier. They will not come to your program. Because of the competition.*”

(Student Health Services Staff)

“*One of the things that the pandemic has taught us is we have to be more open and more inclusive to the schedules of students…. Taking those things into consideration I think will make a huge difference in terms of the audience we reach.*”

(Student Health Services Staff)

This concern was supported by students. When asked about their day-to-day concerns and priorities, focus group participants expressed concerns regarding academic and social responsibilities. While students did express concerns regarding health and HIV risk among their peers, many did not perceive themselves at risk for HIV nor consider HIV/STI prevention a high priority:

“*Like once you go to help center it’s a sign that says only STI testing is like online two days a week. And I feel like that’s like very limiting to people, especially their class schedules and their lifestyles.*”

(Focus group participant)

“*Maybe a year or almost a year ago, that was my top thing that I was always concerned about. I didn’t have many partners, but I wouldn’t say [this city] is like the best place or the community is always small so that was something big on my list about a year, almost a year ago.*”

(Focus group participant)

Although many students did not perceive themselves to be at risk for HIV, they emphasized the importance of expanding HIV prevention programs on campus. They expressed a strong desire for more education, easier access to condoms, and increased availability of HIV testing.

“*So, I think that’s something that definitely should be cleared up especially since we’re in a heavily populated HIV area… and, you know, especially with college students, no matter where, there is going to be a lot of sexual activity on campus. And so really getting that information out and really, fully truly educating people on what it means to be prepared…*”

(Focus group participant)

#### 3.1.8. Navigating Stigma

HIV stigma is a barrier to HIV testing and accessing condoms on campus. Students expressed concerns about maintaining privacy when it comes to HIV testing. While some students expressed willingness to engage in HIV testing and condom distribution activities, concerns about stigmatization were a deterrent for students engaging in HIV prevention activities in a public setting:

“*I think some people may actually just avoid trying to go inside clinics, just because like they might think I don’t want nobody to see me going in here or whatever have you.*”

(Focus group participant)

Students also expressed hesitation to discuss HIV with others due to fear of judgement:

“*Some people…they think that ‘oh, you’re dirty.’ or ‘you were messing around with a lot of people…. I guess there’s such a big stigma around it and everybody still has that mindset of it, that it’s hard to know who you can go to without feeling like you’re going to get judged.*”

(Focus group participant)

#### 3.1.9. Tailored Implementation Plans

The tailored implementation plans we designed for campuses focused heavily on engaging peers to provide educational information and increasing the capacity to deliver HIV testing and condom distribution through leveraging existing external partnerships and developing new ones. These plans were developed using a two-step process. First, individual meetings were conducted with leadership from each campus to present key findings. Following these initial meetings, implementation planning meetings were held to develop an intervention implementation plan for each campus. To support this process, the study staff created implementation planning guides to assist with developing plans for each component (HIV testing, condom distribution and educational activities) of Take CHARGE. These guides addressed resource gaps and utilized facilitators identified in the pre-implementation data. The guides also gathered information on the budget, key campus contacts, community partners, and implementation objectives and strategy. For peer education activities, the guides also included details on developing a job description, compensation, onboarding, and the supervision and reporting structure. Each campus received USD 30,000 to implement the intervention.

### 3.2. Post-Implementation

Post-implementation data collection provided an opportunity to reassess barriers and facilitators identified in the pre-implementation phase, while also identifying new challenges and enablers that occurred during implementation. Key themes that emerged as impacting implementation included institutional culture, external partnerships, student access to resources, internal communication, and peer educator recruitment and retention. Table 4 outlines the pre- and post-implementation themes categorized by CFIR domain.

#### 3.2.1. Institutional Culture

Despite pre-implementation interviewees’ suggestions that a conservative culture would not impact implementation, as well as students expressing a desire for more information and services, post-implementation interviews suggested that there was some division among students and administration regarding the discussion of sexual health and HIV prevention on campus. These challenges highlight the pervasiveness of HIV-related stigma.

“*There are students who are all for it and then you also see a 180-degree difference from that point of students who very much believe that you shouldn’t be talking about it. I don’t want to see anything about it. I don’t want to be involved. There is a huge variation among students. I would have to say probably also among the staff and faculty.*”

(Campus Liaison)

Some key informants reported challenges with support for HIV prevention efforts— specifically condom distribution—due to stigma associated with HIV, sexual behavior, and the image of the institutions:

“*Resistance was around condoms and distributing condoms.... I think they got some resistance with regards to how open we were …. If we’re providing them a mechanism to do what they’re doing safely, I don’t think that there should be any kind of moral consideration given because what we need to do is to stop the spread of these diseases.*”

(CBO Staff)

#### 3.2.2. External Partnerships

External partnerships were a key capacity building strategy implemented across all schools. Key informants reported few barriers related to external partnerships with partnering CBOs and local public health departments. However, COVID-19 restrictions limited the ability of CBOs to come to campus to conduct HIV testing and educational programming.

External partnerships and support from Take CHARGE personnel were identified as key components of successful implementation. External partners provided experienced personnel to conduct training for peer health educators, supplied condoms and HIV testing supplies, reduced the burden on limited staff members, and provided technical assistance throughout implementation.

“*The partnership with DPH, the [local] Health Department has been amazing, and I couldn’t do this without them. I’m very, very happy that they were able to help out.*”

(Campus Liaison)

#### 3.2.3. Increased Student Access to Health Resources 

Key informants generally regarded the intervention as a success. Through the establishment of external partnerships, peer health education, and condom distribution strategies, all key informants reported an increase in HIV prevention programming on their campuses:

“*With Project Take Charge I was able to increase that number of testing opportunities for the students.*”

(Campus Liaison)

Additionally, key informants suggested that the implementation had a direct impact on the students that participated in the intervention. Key informants reported that peer health educators effectively addressed HIV-related misinformation among their peers:

“*I would say the students are already engaged and evolved. They have a better sense of dispelling the rumors and the misconceptions.*”

(Campus Liaison)

#### 3.2.4. Internal Communication

Internal communication failures were described as prominent barriers to implementation. While there were faculty, staff, and administration that were in favor of implementing HIV prevention programming on campus, several key informants mentioned the lack of communication between departments. Consequently, key stakeholders were often unaware of other related activities or institutional processes occurring on campus:

“*… The internal communication is absolutely a problematic area at [the college]. There [are] not too many opportunities where right-hand actually knows what the left hand is doing. The lack of internal communication and coordination, it’s not only, again, [Take Charge], but that is a problem, and that actually hinders what it is that we are trying to do.*”

(Administration)

Key informants suggested that departments working in silos to engage with students decreased the number of students that could be served by the intervention and hindered attempts to increase HIV prevention programming on campus:

“*To be honest with you, that connection, to me what I hear all the time, is absolutely broken. It is not specific to the areas related to [Take Charge] services or support systems that [student health] can provide possibly related to topics and areas that [Take Charge] covers, but even in the mental health, to this day, I have no idea how our students actually access [student health] services.*”

(Campus Liaison)

Additionally, social media strategies played a crucial role in directly communicating program activities to students. While some key informants felt this approach was ineffective, others expressed challenges navigating institutional marketing and communication policies:

“*In terms of the social media, as I noted to you earlier, that falls under the communication, and [the college] has a very pretty strict or strong reign on what gets put out there by student organizations. Talking to the communication and then making sure what can and cannot be put out there, who owns the logo, and how all of that works out. That was one of the headaches, to be honest with you.*”

(Campus Liaison)

#### 3.2.5. Peer Educator Recruitment and Retention

Key informants reported challenges with recruiting and retaining peer educators. They noted that many students were either not interested in participating, or had conflicting responsibilities that took priority over involvement in student health and wellness activities. Additionally, when students were identified for peer educator training, campus liaisons reported delays in onboarding processes, which often led to students losing interest or committing to other responsibilities:

“*The peer educators were identified early on, probably around maybe October of the latest, but they’re just being started. The onboarding process was completed for two of them, and that took it to the end of January. The length of time was time-consuming.*”

(Campus Liaison)

## 4. Discussion

In this qualitative study, we aimed to assess the barriers and facilitators to implementing the Take CHARGE project at HBCUs. We used a two-phase approach that included conducting key informant interviews both before and after project implementation. Additionally, we held student focus groups before the implementation to gather baseline data. In the initial phase, the study identified several anticipated barriers and facilitators, focusing on elements such as institutional culture and external partnerships, which were important for understanding the factors that could influence the project’s success. Following the implementation, post-intervention data collection was carried out to assess the ongoing challenges and support encountered during the implementation of the intervention. The difficulties encountered in peer educator recruitment and retention and internal communication, which were not initially anticipated, were identified as potential barriers. 

Interestingly, despite pre-implementation predictions suggesting that a conservative institutional environment would not pose a major obstacle, post-implementation revealed a different scenario. There was a notable division among the administration concerning the appropriateness of actively promoting sexual health and HIV prevention on campus. This division reflects findings from a study by Warren-Jeanpierre and Jones [12], which indicated that conservative campus atmospheres might impede HIV prevention efforts at HBCUs. They found these cultural mindsets could also affect how students perceive and participate in HIV prevention approaches [12]. Additionally, the conservative culture of HBCUs tends to promote heteronormativity, which may marginalize other experiences and perspectives and promote secrecy, inhibiting open discussions of sexual health on these campuses [10,12]. While stigma may contribute to a cultural reluctance to discuss sexuality in more conservative contexts, studies have identified effective strategies for engaging African American faith-based institutions in HIV prevention [38,39,40]. For instance, Nunn et al. [38] shared lessons learned from an HIV/AIDS testing campaign developed in partnership with 40 African American faith-based institutions in Philadelphia, Pennsylvania. The campaign employed media engagement and diverse messaging about HIV testing, treatment, and reducing the number of sexual partners. 

Furthermore, internal communication failure was described as a significant obstacle to implementing HIV prevention programs. Although some faculty, staff, and administration were supportive of such initiatives, a lack of interdepartmental communication was frequently cited by key informants as a major impediment. This resulted in reduced student participation in the programs, thereby limiting the potential reach and effectiveness of HIV prevention efforts.

Despite these challenges, the three institutions involved in the study successfully leveraged external partnerships and the enthusiasm of student peer educators to effectively deliver the Take CHARGE HIV prevention services. Our findings are consistent with those reported by He et al. (2020) [41] and Birdthistle et al. (2018) [42], who also noted the positive impacts of engaging community members, including youth ambassadors, in HIV prevention efforts. These strategies not only increased HIV testing and treatment, but also contributed to a significant reduction in HIV incidence. Additionally, the systematic review and meta-analysis by He et al. (2020) [41] emphasized the effectiveness of peer-based interventions in HIV prevention. Specifically, they found that peer education contributed to a 36% reduction in HIV infection rates among high-risk populations (OR: 0.64; 95%CI: 0.47–0.87). Furthermore, peer education significantly increased HIV testing (OR = 3.19; 95%CI: 2.13, 4.79) and condom usage (OR = 2.66, 95% CI: 2.11–3.36), highlighting the role of peer-led interventions in enhancing HIV-related knowledge, particularly regarding transmission routes.

Moreover, our study identified the cost of implementing HIV prevention activities as another barrier for all institutions. This finding aligns with previous studies [43,44] which also identified the associated cost as the primary barrier to HIV testing. For instance, a study of New England college health centers revealed that medical directors identified financial barriers as a significant concern. These barriers included the cost to students and a lack of resources and support services needed to promote and provide testing [43]. Similarly, a qualitative study by Lin et al. [44] demonstrated that students face various obstacles when it comes to HIV testing. These obstacles include a lack of information, economic costs, moral judgment, and concerns about potential repercussions for their social standing [44].

Pre-implementation focus group discussions with students highlighted several areas of alignment and conflict when compared to the perspectives of faculty, staff, and administration gathered from key informant interviews. On the one hand, students acknowledged that competing priorities sometimes conflicted with their participation in HIV prevention programming, which aligned with key informants’ acknowledgement of the role student priorities play in implementing HIV prevention programming. Both groups expressed a shared desire for more robust HIV prevention efforts. However, there were notable points of conflict between the two perspectives. While students lamented the lack of sufficient HIV prevention programming, key informants felt that they were well positioned to address these service gaps. Furthermore, although both students and key informants acknowledged the conservative attitudes toward sexual health at their institutions, students were more vocal about the impact of HIV stigma and its effect on student engagement in HIV prevention activities, especially HIV testing. This concern was less prominent in the key informant interviews. 

Our study also revealed that although students expressed concerns regarding health and HIV risks among their peers, many did not perceive themselves to be at risk for HIV, nor did they prioritize HIV/STI prevention. Likewise, an analysis of an online survey targeting African American undergraduate students at HBCUs assessed their HIV/AIDS knowledge and behaviors. This survey found that 79% of the respondents considered themselves to be at low risk for HIV infection, and notably, 64% of those who had engaged in sexual activity with two or more partners had not used a condom during their last sexual encounter [45].

A key limitation of this study was the inability to conduct post-implementation focus groups due to insufficient student recruitment at each site. Future research should prioritize gathering student perspectives on the impacts of similar HIV prevention programming, as their insights would provide valuable feedback on how such interventions influence student attitudes and engagement with available campus resources.

## 5. Conclusions

As HBCUs are uniquely positioned to support HIV interventions in the South [18], Take CHARGE illustrates opportunities for increasing HIV prevention programming on the campuses of HBCUs and PBIs through increased capacity-building, culturally tailored implementation strategies, and concerted efforts to combat sexual health stigma among students and college administrators. 

Intervening at the structural level on these campuses requires assessing their infrastructure and capacity and encouraging HBCUs and other PBIs to leverage existing partnerships and forge new ones to create effective and sustainable HIV prevention interventions. Both pre- and post-implementation data indicated that external partnerships were instrumental in the successful implementation of Take CHARGE. These partnerships provided supplemental expertise for culturally competent health education and training, and supported condom distribution and HIV testing, activities that were constrained by limited personnel and budgets. Moreover, to effectively implement HIV prevention programming, there is a clear need to increase communication across the institution through the involvement of campus stakeholders (administration, faculty, staff, and students) and promote evidence-based strategies that align with institutional priorities and policies. Including all relevant stakeholders in the design and implementation processes may help prevent delays and inefficiencies. Strengthening collaboration across departments will optimize the impact of HIV prevention initiatives on college campuses.

The findings from Take CHARGE may serve as a model for future HIV prevention efforts at HBCUs and PBIs. By addressing the identified barriers and building on the facilitators, institutions can enhance their capacity to deliver effective, culturally relevant HIV prevention services and, ultimately, contribute to reducing the disproportionate burden of HIV among Black Americans.

## Figures and Tables

**Table 1:** Key Informant Characteristics.

Characteristic		*N*	%
Total		23	100
Race	Black	22	96%
	Asian/pacific islander	1	4%
Sex Assigned at Birth	Male	8	35%
	Female	15	65%
Role	Faculty	4	17%
	Student health services	9	39%
	Administration	8	35%
	CBO Staff	2	9%

**Table 2:** Focus group characteristics.

Characteristics		N	%	Mean	s.d.
		40	100		
Age				20.18	1.52
Sex Assigned at Birth	Male	22	55		
	Female	18	45		
Classification	Freshman	8	20		
	Sophomore	9	22.5		
	Junior	13	32.5		
	senior	9	22.5		
	graduate student	1	2.5		

**Table 3:** CFIR domains.

Adapted CFIR Domains	Description	Subthemes	Data Source
**Intervention Characteristics**			
Relative Advantage	Perceptions of the advantage of implementing the innovation versus an alternative solution.	Perceived need for the intervention, increased student access to resources	Key informant interviews
Cost	Costs of the intervention and costs associated with implementing the intervention, including investment, supply, and opportunity costs.	Cost concerns	Key informant interviews
**Outer Setting**			
Student needs and resources	Identifying the specific needs and resources of students aged 18–24 related to HIV and substance use prevention. Includes barriers, knowledge, attitudes, behaviors, and preferences.	Student access to resources, student priorities, peer educator recruitment and retention, navigating stigma	Key informant interviews, Focus groups
Cosmopolitanism	The extent to which institutions collaborate with external organizations to facilitate the implementation of HIV and substance use prevention. Includes challenges associated with these partnerships.	Personnel, External partnerships	Key informant interviews, Focus groups
**Inner Setting**			
Structural characteristics	The social architecture, age, maturity, and size of an institution.	Personnel	Key informant interviews
Available resources	The level of resources dedicated for implementation and ongoing operations, including money, training, education, and physical space.	Dedicated campus resources	Key informant interviews
Network and communication	The nature and quality of social networks and the nature and quality of informal and formal communicators within an organization	Internal communication	Key informant interviews
Culture	The norms, values, and basic assumptions of participating campuses. Includes attitudes and stigmas that may impact the implementation or uptake of HIV and substance use prevention programming	Institutional culture, navigating stigma	Key informant interviews, focus groups

**Table 4:** Pre and Post Implementation Themes.

Adapted CFIR Domains	Pre-Implementation Sub-Themes	Post-Implementation Sub Themes
**Intervention Characteristics**		
Relative Advantage	Perceived need for the intervention	Increased student access to resources
Cost	Cost Concerns	
**Outer Setting**		
Student needs and resources	Student access to health resources, student priorities, navigating stigma	Increased student access to resources, peer educator recruitment and retention
Cosmopolitanism	Personnel	External partnerships
**Inner Setting**		
Structural Characteristics	Personnel	
Available Resources	Dedicated campus resources	
Network and Communications		Internal communication
Culture	Institutional culture, navigating stigma	Institutional culture

## Data Availability

The data presented in this study are not available because the data are qualitative and the words, situations presented, and phrasing of the wording can potentially identify participants and compromise confidentiality.
